# Exploring the effects of lifestyle on breast cancer risk, age at diagnosis, and survival: the EBBA-Life study

**DOI:** 10.1007/s10549-020-05679-2

**Published:** 2020-05-20

**Authors:** Trygve Lofterød, Hanne Frydenberg, Vidar Flote, Anne Elise Eggen, Anne McTiernan, Elin S. Mortensen, Lars A. Akslen, Jon B. Reitan, Tom Wilsgaard, Inger Thune

**Affiliations:** 1 Department of Oncology, Oslo University Hospital, Oslo, Norway; 2 Faculty of Health Services, Institute of Community Medicine, University of Tromsø, Tromsø, Norway; 3 Public Health Sciences Division, Fred Hutchinson Cancer Research Center, Seattle, USA; 4 Department of Pathology, University Hospital of North Norway, Tromsø, Norway; 5 Department of Clinical Medicine, Centre for Cancer Biomarkers CCBIO, University of Bergen, Bergen, Norway; 6 Department of Pathology, Haukeland University Hospital, Bergen, Norway; 7 Faculty of Health Services, Institute of Clinical Medicine, University of Tromsø, Tromsø, Norway

**Keywords:** Lifestyle, Breast cancer, Onset, Risk, Survival

## Abstract

**Purpose:**

Whether an unfavorable lifestyle not only affects breast cancer risk, but also influences age at onset of breast cancer and survival, is under debate.

**Methods:**

In a population-based cohort, the Energy Balance and Breast Cancer Aspects throughout life (EBBA-Life) study, a total of 17,145 women were included. During follow-up, 574 women developed invasive breast cancer. Breast cancer cases were followed for an additional 9.1 years. Detailed medical records were obtained. Cox’s proportional hazard regression models were used to study the association between pre-diagnostic lifestyle factors (weight, physical activity, alcohol use, smoking, and hypertension), breast cancer risk, age at diagnosis, and survival.

**Results:**

At study entry, 34.3% of the participating women were overweight and 30.7% were physically inactive. Mean age at breast cancer diagnosis was 58.0 years, and 78.9% of the tumors were estrogen receptor positive. Among menopausal women who did not use hormone therapy and had an unfavorable lifestyle (3–5 unfavorable factors), compared with women who had a favorable lifestyle, we observed a twofold higher risk for postmenopausal breast cancer (hazard ratio [HR] 2.13, 95% confidence interval [CI] 1.23–3.69), and they were 3.4 years younger at diagnosis (64.8 versus 68.2 years, *P* = 0.032). Breast cancer patients with an unfavorable lifestyle, compared with patients with a favorable lifestyle, had almost a two times higher overall mortality risk (HR 1.96, 95% CI 1.01–3.80).

**Conclusions:**

Our study supports a healthy lifestyle improving breast cancer prevention, postponing onset of disease, and extending life expectancy among breast cancer patients.

**Electronic supplementary material:**

The online version of this article (10.1007/s10549-020-05679-2) contains supplementary material, which is available to authorized users.

## Introduction

A global increase in breast cancer incidence has been observed, with incidence rates being almost fourfold higher in the developed than in the less-developed countries, emphasizing that large differences in lifestyle may have an important role to play [1]. Of note, the rates of estrogen receptor (ER)-positive breast cancer incidence are increasing [2], suggesting that an estrogen-dependent mechanism links unfavorable lifestyle to this most common breast cancer subtype. Furthermore, life expectancy for women who have survived breast cancer has been observed to be shorter compared with women in the general population [3], but whether lifestyle factors play a key role in relation to breast cancer survival remains ambiguous.

The association between body composition, including body weight and breast cancer risk, is well known. High Body Mass Index (BMI) has been positively associated with postmenopausal breast cancer [4, 5], but most studies report that excess body weight is inversely related to premenopausal breast cancer risk [6]. Obesity has also been associated with reduced breast cancer survival [7]. A physically active lifestyle compared with a sedentary lifestyle has been observed to reduce both pre- and postmenopausal breast cancer risk overall [8, 9]. The association between physical activity and breast cancer survival is still being debated [9]. The most consistent dietary risk factor for breast cancer is alcohol [10, 11]. Alcohol intake has been associated with higher estradiol levels and higher mammographic density [12], and both pre- and postmenopausal breast cancer risk [13, 14]. Recent meta-analysis observed a positive association between hypertension and breast cancer risk [15, 16]. Moreover, a new cohort study reported a positive association between hypertension and breast cancer mortality after adjustments [17], supporting hypertension as an independent risk factor linked to breast cancer. Tobacco smoking possesses potential mammary carcinogens [18], and recently tobacco use showed an increased risk for ER-positive breast cancer [19].

Possible biological mechanisms operating for most of these lifestyle factors may be mediated by adipose tissue, with low-grade chronic inflammation creating an environment that promotes breast cancer development and growth [20–22]. Another potential mechanism linking these factors is via increased estrogen levels, a key component in breast cancer development [9]. In addition, both hypertension and breast cancer development may act through activation of the renin-angiotensin system [23]. The proposed pathophysiological processes related to unfavorable lifestyle factors, e.g., chronic inflammation and insulin resistance, are also established biological mechanisms associated with aging, a known risk factor for cancer development [24]. Moreover, a rising trend within the general population of being overweight appears to parallel a shift to the appearance of cancer at an earlier age [25]; this suggests that obesity and other unfavorable lifestyle factors not only promote tumor growth, but also affect aging and breast cancer onset. Hence, one may hypothesize that lifestyle factors play a role in relation to optimal breast cancer treatment, comorbidity, and breast cancer survival.

The main aim of the present study was, therefore, based on a population-based cohort study, with a high attendance rate and detailed medical and histopathological information, to explore the joint effect of lifestyle factors on breast cancer risk, age at onset, and survival.

## Materials and methods

This population-based cohort, the Energy Balance and Breast Cancer Aspects throughout life (EBBA-Life) study [26, 27], is a substudy of the Tromsø study [28]. A total of 20,619 women, aged > 20 years, participated in the population-based Tromsø study in five waves of almost identical data collection, conducted between 1986 and 2016, carried out 6–7 years apart with an attendance rate of 74.0% [28, 29]. At study entry, all participants completed questionnaire data and sampling of biological specimens, and basic clinical measurements were performed. All data collection was carried out by trained research technicians at each survey.

### Questionnaires

The questionnaires were filled in at home and brought to the study site, where they were checked for completeness and inconsistency. Questionnaires included items about medical history, specific symptoms, dietary habits, lifestyle factors, reproductive factors, and use of medication including antihypertensive drugs and hormone therapy [28].

### Assessment of lifestyle factors and menopausal hormone therapy (MHT)

Height and weight were measured [28], and the BMI (kg/m^2^) was calculated. Physical activity was reported according to type of physical activity over the last 12 months (walking/cycling, recreational sport, strenuous training, or participating in sports competition), duration, and hours of intensity exercise per week. Categories of physical activity were classified as follows: (1) sedentary physical activity: reading/sitting with no participation in recreational sport activities or competitions over the last 12 months; (2) moderate physical activity: walking/cycling at least 4 h a week and/or minimum of 1 h of strenuous physical activity per week (sweating/out of breath); and (3) hard physical activity: participating in strenuous training or sports competitions regularly/several times a week and/or exercise approximately every day. Alcohol intake was reported according to the number of days a month when alcohol was drunk, and has been validated [30]. Smoking was reported and classified as never/past smokers or current smokers. Blood pressure (mmHg) was measured [28]. MHT use was reported and classified as past/present use or never use. Baseline information on MHT use was obtained when the participants first entered the study, and updated MHT use was performed at each new wave between 1986 and 2016.

### Identification of breast cancer cases, breast tumor characteristics, and medical charts

All breast cancer cases were identified through linkage to the Cancer Registry of Norway by using the unique, national, 11-digit identification number [31]. We obtained information on death and emigration from the Cause of Death Registry and the National Population Registry, respectively [32, 33]. Death from breast cancer was coded according to International Classification of Disease (ICD) [34]. We excluded all attendees who had a previous history of cancer, or who emigrated, died, or were diagnosed with cancer within the first year after study entry (*n* = 709). All women with missing information on BMI, physical activity, alcohol use, smoking, blood pressure, or MHT use were excluded (*n* = 2 765). Thus, 17,145 women were included in the final sample. The participants were followed from the date of entry into the study until the date of breast cancer diagnosis, date of emigration, date of death, or end of follow-up (December 31, 2017), whichever event occurred first.

The breast cancer patients’ medical charts were reviewed to obtain detailed clinical data, including breast cancer histological type, grade (1–3), tumor stage (1–4) according to the TNM (tumor, node, metastases) classification, and breast cancer treatment. A total of 574 women were diagnosed with incident invasive breast cancer during follow-up (Online Resource 1). Follow-up after breast cancer diagnosis was calculated from the date of the diagnosis to the date of death, emigration, or end of follow-up.

All breast tumor samples were fixed in 4% buffered formaldehyde before processing and embedding in paraffin. To obtain more complete and updated information on tumor characteristics, most (*n* = 407) of the tissue samples were analyzed on tissue microarrays (TMAs) at the University of Bergen, Norway (Centre for Cancer Biomarkers) [26, 35]. Breast tumor specimens not reanalyzed on TMA blocks (*n* = 167) were evaluated using immunohistochemistry for hormone receptor status and Ki-67, and immunohistochemistry and fluorescence in situ hybridization for human epidermal growth factor receptor-2 (HER2). A subset of the breast cancer cases (*n* = 522) with complete information on receptor status were categorized into three molecularly defined subgroups: (1) ER positive—patients with ER-positive (with or without progesterone (PgR)-positive) and HER2-negative status; (2) HER2 positive—all patients with HER2 overexpression; and (3) triple-negative breast cancer (TNBC)—HER2−, ER-−, and PgR-negative status.

### Statistical methods

To characterize the change in incidence rates during follow-up, crude incidence rates were calculated as new breast cancer cases per 1000 person-years at risk for disease for the total cohort (30–90 years) and within the age groups: ≤ 55 years and > 55 years. Non-linearity in incidence trends was considered and an estimate calculated using a fractional polynomial in Poisson’s regression models. A total of 44 models were estimated to find the best-fitting model to describe trends in breast cancer incidence.

Multivariable Cox’s proportional hazard regression models were used to study whether a pre-diagnostic unfavorable lifestyle, assessed at baseline, was associated with breast cancer risk and mortality. To study the importance of the variation in lifestyle factors independently and in combination, we identified five different modifiable lifestyle-related factors associated with breast cancer development, and categorized each of them into favorable versus unfavorable based on international categorization (WCRF/AICRF, World Health Organization) [36, 37]:

1. *Body composition* favorable—BMI < 25 kg/m^2^ versus unfavorable—BMI ≥ 25 kg/m^2^.
2. *Physical activity* favorable—moderate or more physical activity versus unfavorable—sedentary physical activity.
3. *Alcohol use* favorable—no alcohol or ≤ 1 day a month drinking alcohol versus unfavorable— > 1 day a month drinking alcohol.
4. *Smoking* favorable—no current smoking versus unfavorable—current smoker.
5. *Hypertension* favorable—systolic blood pressure < 140 mmHg, diastolic blood pressure < 90 mmHg, and no antihypertensive medication versus unfavorable—systolic blood pressure ≥ 140 mmHg and/or diastolic blood pressure ≥ 90 mmHg, and/or use of antihypertensive medication.

The participants scored 0 for each favorable lifestyle factor and 1 for each unfavorable one, resulting in a score range of 0–5. We then split these modifiable lifestyle factors into four categories according to the sum of unfavorable lifestyle factors: category 1 (reference): score 0; category 2, score 1; category 3, score 2; category 4, score 3–5. We constructed three separate regression models for each exposure to evaluate overall, premenopausal, and postmenopausal breast cancer as model-specific outcomes. Based on previous studies among women from the same cohort [27], premenopausal status was defined as age ≤ 55 years, and all women aged > 55 years were categorized as postmenopausal. MHT use is a strong risk factor for breast cancer, and therefore we ran separate time-dependent regression analyses according to updated information on MHT use during follow-up. We used a linear regression model to study the association between clustering of unfavorable lifestyle factors and age at diagnosis. One-way analysis of variance (ANOVA) was used to study the differences between number of unfavorable lifestyle factors and breast tumor characteristics.

Based on suggested biological mechanisms influencing these modifiable lifestyle factors and/or breast cancer risk and prognosis, several variables were studied as potential confounders: age (continuous), age at menarche (continuous), and number of live births (continuous). In the final analysis of survival, only age was included as a potential confounder. Breast cancer stage (categorical) did not influence our results, and was not included. A total of 111 breast cancer cases were checked for agreement between subtyping based on immunohistochemistry and TMA. We observed an agreement between these two methods in 93% of the breast cancer cases (*κ* = 0.76). All the tests were two sided and the statistical significance was defined by *P* < 0.05. Statistical analyses calculating crude incidence of new breast cancer cases were conducted using STATA 14 (StataCorp, College Station, TX, USA). All other statistical analyses were conducted using SPSS 21.0 (IBM Corporation, Armonk, NY, USA).

## Results

**Fig. 1:**
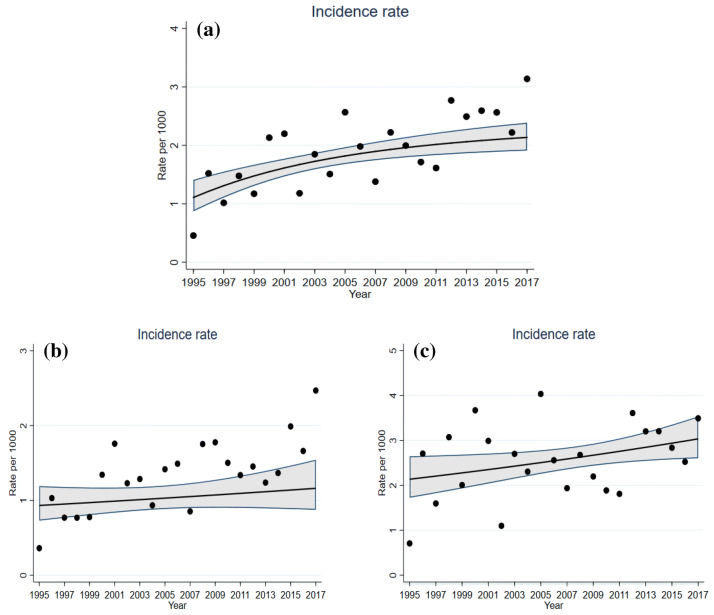
Breast cancer incidence rate in the period between 1995 and 2017 among: **a** women aged 30–90 years, **b** women aged 30–55 years, and **c** women aged 55–90 years

Among the 17,145 women included at study entry, 34.3% were overweight, 30.7% physically inactive, and 58% consumed alcohol > 1 day/month. We observed a 93% increase in overall age-adjusted breast cancer incidence through the period from 1995 to 2017 (incidence rate ratio 1.93, 95% CI 1.42–2.62) (Fig. 1). Mean age at breast cancer diagnosis was 58.0 (range 31.2–92.0) years, and 78.9% of the breast tumors were ER positive (Table 1). The distribution of the molecularly defined breast cancer subtypes was as follows: ER positive (69.5%, *n* = 362), HER2 positive (16.3%, *n* = 85), and TNBC (14.2%, *n* = 75) (Online Resource 2).

**Table 1:** Characteristics of women without breast cancer (non-cases) and breast cancer patients (cases): the EBBA-Life study (1986–2017)

	All women (*n* = 17,145)^a^	Non**-**cases (*n* = 16 571)^a^	Breast cancer cases (*n* = 574**)**^a^
	Mean (SD)/%	Mean (SD)/%	Mean (SD)/%
Characteristics at study entry			
Age at attendance, years	41.7 (13.8)	41.7 (13.8)	41.3 (12.4)
Follow-up, years	20.1 (10.0)	20.2 (11.1)	16.7 (7.85)
Overall mortality rate, %	14.3	13.9	26.3
Reproductive factors			
Number of children	2.11 (1.36)	2.11(1.36)	2.03 (1.26)
Age at menarche, years	13.2 (1.47)	13.2 (1.47)	13.1 (1.43)
Clinical variables			
Height, cm	164 (6.58)	164 (6.57)	165 (6.06)
BMI, kg/m^2^	24.4 (4.34)	24.4 (4.36)	23.7 (3.63)
Systolic blood pressure, mmHg	122 (19,0)	122 (19.1)	122 (17.6)
Diastolic blood pressure, mmHg	72.4 (10.9)	72.3 (11.0)	73.3 (10.7)
Lifestyle factors/Comorbidity			
MHT users^b^, %	4.5	4.5	4.2
Blood pressure treatment, %	4.99	5.02	4.08
Overweight^c^, %	34.3	34.6	27.1
Physically inactive^d^, %	30.7	30.6	32.3
Alcohol consumption > 1 day/month, %	58.1	58.3	52.7
Current smokers, %	41.0	40.9	44.2
Hypertension^e^, %	18.2	18.2	18.0
Characteristics among breast cancer cases			
Age at diagnosis, years			58.0 (11.7)
Observation time after diagnosis, years			9.11 (6.91)
Cancer-specific mortality rate, %			9.8
Tumor characteristics			
Histological subtype, %			
Invasive carcinoma NST			79.7
Invasive lobular carcinoma			13.1
Others			7.2
Tumor size, mm			23.0 (18.8)
Stage, %			
1–2			82.9
3			13.9
4			3.2
Histological grading, %			
1			28.7
2			46.9
3			24.4
Estrogen receptor positive, %			78.9
Progesterone receptor positive, %			59.8
HER2 positive, %			16.3
Ki-67, %			20.6 (17.8)
Treatment			
Chemotherapy, %			36.0
Endocrine therapy, %			40.9
Radiation therapy, %			78.5

*BMI* Body Mass Index (kg/m^2^), *HER2* human epidermal growth factor receptor-2, *MHT* menopausal hormone therapy, *NST* no special type, *SD* standard deviation

^a^Numbers may vary due to missing information

^b^MHT users at baseline

^c^Overweight defined as BMI ≥ 25 kg/m^2^

^d^Physically inactive: reading/sitting with no participation in recreational sport activities or competitions during the last 12 months

^e^Systolic blood pressure ≥ 140 mmHg and/or diastolic blood pressure ≥ 90 mmHg, and/or use of antihypertensive medication

### Pre-diagnostic lifestyle factors: breast cancer risk

Among all the women, those drinking alcohol > 1 day/month versus those drinking alcohol 0–1 day/month had a 31% higher overall breast cancer risk (hazard ratio [HR] 1.31, 95% CI 1.10–1.56). Current smoking versus no/previous smoking was associated with a 30% decreased premenopausal breast cancer risk (HR 0.70, 95% CI 0.53–0.91) (Table 2).

**Table 2:** Age-adjusted hazard ratios for incident breast cancer overall and according to menopausal status and MHT use

	All breast cancers		Premenopausal breast cancer		Postmenopausal breast cancer			
					MHT non-users		MHT users	
	*n* = 574	HR (95% CI)	*n* = 246	HR (95% CI)	*n* = 237	HR (95% CI)	*n* = 91	HR (95% CI)
Modifiable lifestyle factors								
Overweight^a^		0.92 (0.75–1.12)		0.88 (0.64–1.23)		1.16 (0.86–1.55)		0.88 (0.53–1.33)
Physical inactivity^b^		1.07 (0.90–1.29)		1.20 (0.90–1.59)		1.10 (0.81–1.48)		0.79 (0.51–1.23)
Alcohol use^c^		1.31 (1.10–1.56)		1.05 (0.80–1.38)		1.57 (1.20–2.06)		1.18 (0.82–1.71)
Cigarette smoking^d^		0.94 (0.79–1.12)		0.70 (0.53–0.91)		1.16 (0.89–1.59)		1.11 (0.77–1.16)
Hypertension^e^		0.97 (0.67–1.13)		1.17 (0.72–1.90)		0.85 (0.60–1.21)		1.00 (0.60–1.68)
Number of unfavorable lifestyle factors								
0 (ref)	62	1.00	34	1.00	17	1.00	11	1.00
1	200	1.41 (1.04–1.78)	91	1.05 (0.71–1.55)	71	1.66 (0.97–2.83)	38	1.58 (0.86–2.92)
2	176	1.26 (0.93–1.71)	79	0.90 (0.60–1.34)	72	1.72 (1.01–2.93)	26	1.29 (0.67–2.45)
3–5	136	1.34 (0.97–1.85)	42	0.83 (0.53–1.31)	77	2.13 (1.23–3.69)	17	1.22 (0.60–2.52)
*P*_trend_		0.348		0.222		0.011		0.999

Cox proportional hazard model

Adjusted to age, age at menarche, and number of live births

*CI* confidence interval, *HR* Hazard ratio, *MHT* menopausal hormone therapy, *n* number of cases, *ref* reference

^a^Overweight defined by BMI ≥ 25 kg/m^2^

^b^Physical inactivity defined by reading/sitting with no participation in recreational sport activities or competitions during last 12 months

^c^Alcohol use defined by > 1 day of alcohol use per month

^d^Current cigarette smoking

^e^Hypertension defined by systolic blood pressure > 140 mmHg and/or diastolic blood pressure > 90 mmHg, and/or use of antihypertensive medication

Among women who were MHT non-users, those with three to five unfavorable lifestyle factors versus those with no unfavorable lifestyle factors had more than a twofold increased risk of developing postmenopausal breast cancer (HR 2.13, 95% CI 1.23–3.69) (Table 2). No clear association was observed between clustering of unfavorable lifestyle factors and premenopausal breast cancer risk (Table 2).

After stratification by breast cancer molecular subtype, women with three to five unfavorable lifestyle factors versus no unfavorable lifestyle factors had a 43% increased ER-positive breast cancer risk (HR 1.43, 95% CI 0.97–2.10, *P*_trend_ = 0.096). Among postmenopausal women who were MHT non-users, those with three to five unfavorable lifestyle factors versus no unfavorable lifestyle factors had a 142% increased risk for ER-positive postmenopausal breast cancer (HR 2.42, 95% CI 1.27–4.63) (Online Resource 3). No association was observed between clustering of unfavorable lifestyle factors and HER2-positive cancer and TNBC overall (Online Resource 2).

### Pre-diagnostic lifestyle factors: age at diagnosis

Among MHT non-users who developed postmenopausal breast cancer, women with three to five unfavorable lifestyle factors were 3.4 years younger at diagnosis than those with no unfavorable lifestyle factors (64.8 years versus 68.2 years, *P* = 0.032) (Table 3). Furthermore, these MHT non-users with three to five unfavorable lifestyle factors had larger tumors than those with no unfavorable lifestyle factors (26.3 versus 12.3 mm, *P* = 0.023). Other tumor characteristics were not statistically differently distributed throughout the four categories of unfavorable lifestyle factors (Online Resource 4).

**Table 3:** Age at diagnosis among breast cancer patients according to number of unfavorable lifestyle factors by menopausal status at diagnosis and MHT use

	All breast cancers		Premenopausal breast cancer		Postmenopausal breast cancer			
					MHT non-users		MHT users	
	*n* = 574	Age at diagnosis (SE)	*n* = 246	Age at diagnosis (SE)	*n* = 237	Age at diagnosis (SE)	*n* = 91	Age at diagnosis (SE)
Number of unfavorable lifestyle factors^a^								
0	62	58.8 (0.97)	34	49.2 (0.80)	17	68.2 (1.37)	11	63.0 (1.53)
1	200	58.5 (0.54)	91	47.5 (0.48)	71	66.7 (0.68)	38	65.1 (0.81)
2	176	57.4 (0.57)	79	46.6 (0.52)	72	65.7 (0.67)	26	66.0 (0.97)
3–5	136	58.0 (0.67)	42	48.6 (0.72)	77	64.8 (0.68)	17	65.6 (1.23)
*P*^b^		0.517		0.585		0.032		0.198

Linear regression model

Covariates: Age at study entry

*CI* confidence interval, *MHT* menopausal hormone therapy, *n* number of cases, *SE* standard error

^a^Unfavorable lifestyle factors: overweight, physical inactivity, alcohol use, smoking, and hypertension

^b^*p* value reflecting difference between no unfavorable lifestyle factors and three to five unfavorable lifestyle factors

### Pre-diagnostic lifestyle factors: overall mortality and breast cancer-specific mortality

**Fig. 2:**
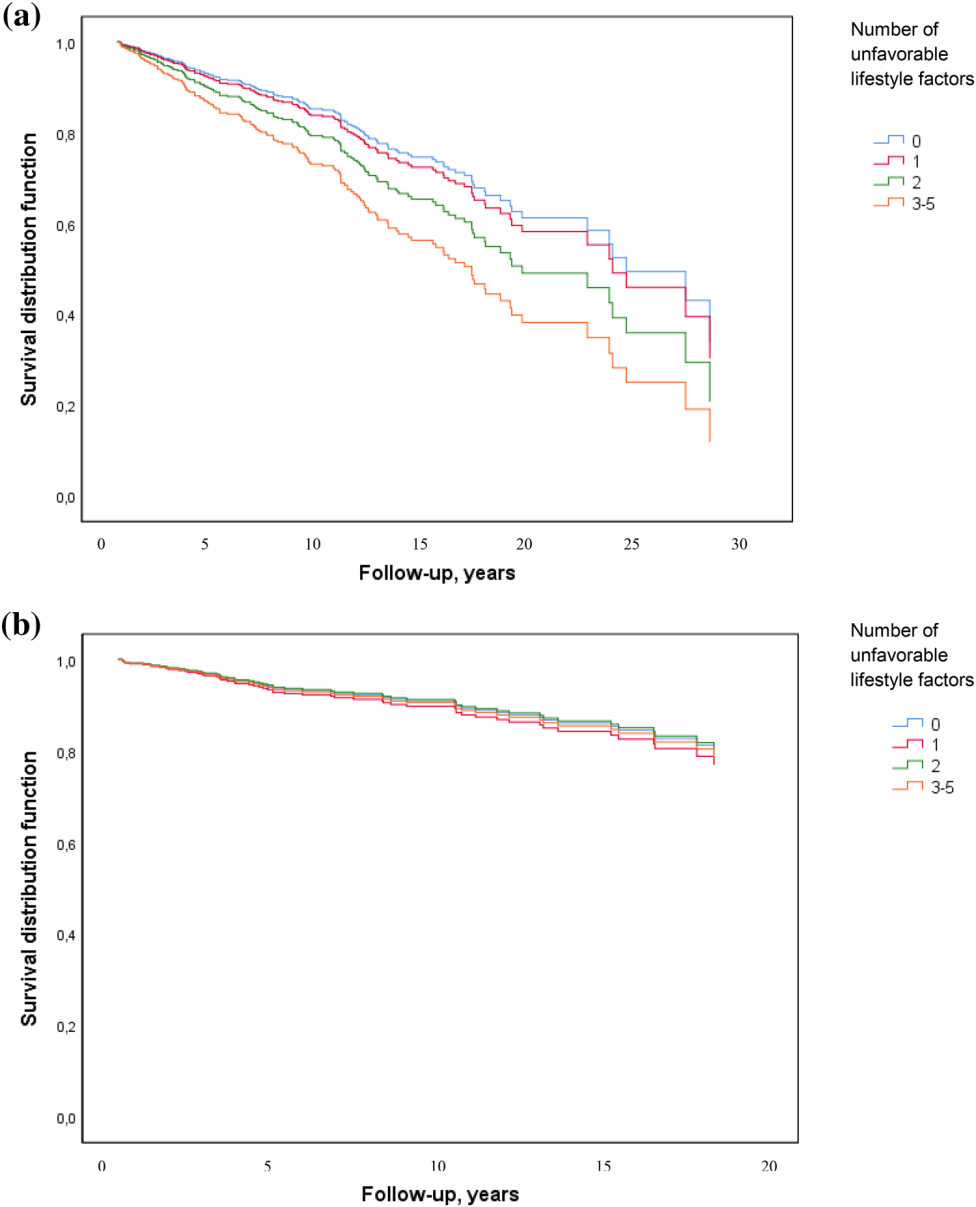
Women diagnosed with breast cancer in the EBBA-Life study (*n* = 574). Number of unfavorable lifestyle factors and **a** overall mortality, and **b** breast cancer mortality Adjusted for age

Among all breast cancer cases combined, we observed a 96% increased overall mortality risk for those breast cancer cases with three to five unfavorable lifestyle factors versus no unfavorable lifestyle factors (HR 1.96, 95% CI 1.01–3.80) (Table 4, Fig. 2). A positive trend between clustering of unfavorable lifestyle factors and overall mortality was observed among premenopausal breast cancer patients (*P*_trend_ = 0.020) (Table 4). Among postmenopausal women, we observed a suggestively inverse trend between clustering of unfavorable lifestyle factors and breast cancer mortality (*P*_trend_ = 0.050) (Table 4).

**Table 4:** Age-adjusted hazards ratios between lifestyle and overall and breast cancer mortality by menopausal status

	All breast cancer cases (*n* = 574)			Premenopausal breast cancer (*n* = 245)			Postmenopausal breast cancer (*n* = 329)		
	Cases (*n*)	Overall mortality (150 deaths)	Breast cancer mortality (56 deaths)	Cases (*n*)	Overall mortality (55 deaths)	Breast cancer mortality (26 deaths)	Cases (*n*)	Overall mortality (95 deaths)	Breast cancer mortality (30 deaths)
		HR (95% CI)	HR (95% CI)		HR (95% CI)	HR (95% CI)		HR (95% CI)	HR (95% CI)
Number of unfavorable lifestyle factors									
0 (ref)	62	1.00	1.00	34	1.00	1.00	28	1.00	1.00
1	200	1.10 (0.57–2.15)	1.14 (0.46–2.82)	91	0.94 (0.34–2.59)	0.90 (0.23–3.40)	109	1.28 (0.53–3.13)	1.37(0.39–4.79)
2	176	1.45 (0.75–2.82)	0.97 (0–37–2.49)	79	1.71 (0.63–4.61)	1.21 (0.32–4.62)	98	1.08 (0.44–2.70)	0.56 (0.14–2.26)
3–5	136	1.96 (1.01–3.80)	1.05 (0.39–2.81)	42	2.16 (0.77–6.06)	1.78 (0.44–7.12)	94	1.39 (0.57–3.39)	0.47 (0.11–1.96)
*P*_trend_		0.005	0.874		0.020	0.260		0.553	0.050

Multivariable Cox proportional hazard regression models

Adjusted for age (continuous)

*CI* confidence interval, *HR* Hazard ratio, *n* number of cases, *ref* reference

## Discussion

In this population-based cohort we observed, over 20 years of follow-up, a 93% increased breast cancer incidence. Furthermore, among women who had a clustering of pre-diagnostic unfavorable lifestyle factors, compared with women who had a favorable lifestyle, we demonstrated a twofold increase in postmenopausal breast cancer risk. This increased breast cancer risk was restricted to MHT non-users, and confined to the most common breast cancer subtype: ER-positive breast tumors. Moreover, postmenopausal women with a pre-diagnostic unfavorable lifestyle were 3.4 years younger at breast cancer diagnosis compared with women who had a favorable lifestyle. Our study also demonstrated that a pre-diagnostic unfavorable lifestyle was associated with almost a two times higher overall mortality risk among women with breast cancer.

Our observed results extend previous studies, but are also in part supported by others [38, 39]. The steady increase in breast cancer incidence over the last three decades observed in our cohort is in line with findings in comparable populations [1]. Of note, an increased breast cancer incidence observed in the present study is unlikely to have been influenced by any breast cancer-screening program introduced many years ahead [40].

Our findings of a dose–response association between a joint combination of lifestyle factors and breast cancer risk with a twofold increase in postmenopausal breast cancer risk among women with an unfavorable lifestyle are supported [41–44]. In a large prospective study including mainly postmenopausal women [45], adherence to dietary guidelines [46] was associated with a decreased breast cancer risk, in particular ER-positive breast cancer tumors. In the Nurse’s Health Study cohort, a decreased postmenopausal breast cancer risk was observed, especially for ER-positive breast cancer, among women with the lowest weight gain, no alcohol consumption, high physical activity level, and had no MHT use [47]. Moreover, postmenopausal women who participated in the Norwegian Breast Cancer Screening Program, self-reported lifestyle factors were associated with increased risk for ER-positive luminal A-like and luminal B-like HER2-positive breast cancer [48]. Of note, MHT use has been documented as a strong independent risk factor for breast cancer development [49]. This underlines the importance of investigating the clustering of modifiable lifestyle factors stratified by MHT users and non-users [49–51]. However, the well-defined interaction between obesity, physical inactivity, and breast cancer among MHT non-users and not among MHT users is not generally present for alcohol [52] and smoking [53]. It is of interest that, in a recent study among women participating in the UK Biobank study, a healthy lifestyle, including diet, physical activity, smoking, alcohol intake, and body composition, was observed to attenuate the impact of genetic factors on invasive breast cancer risk [54]. In our age-adjusted regression analysis presenting the lifestyle factors, smoking was inversely related to premenopausal breast cancer risk, in contrast to other studies [55]. Our observation may be a chance finding, and based on recent observations and potential biological mechanisms operating, we chose to include smoking as a lifestyle-associated breast cancer risk factor.

To our knowledge, our results showing an association between an unfavorable lifestyle and earlier age at diagnosis of sporadic breast cancer have not previously been reported in a population-based cohort study. However, in a recent study weight gain was associated with earlier age at diagnosis [56]. Moreover, one early study showed that among *BRCA1*/*BRCA2* mutations carriers, physical exercise and healthy weight were associated with delayed age at breast cancer onset [57]. Hence, previous results and our results suggest that an unfavorable lifestyle may promote tumor growth and alter breast cancer onset in both BRCAI/II mutation carriers and in those at risk of sporadic breast cancer.

In our study, we observed that a pre-diagnostic unfavorable lifestyle was associated with an almost two times higher overall mortality. An association between obesity and more advanced stage and higher grade at diagnosis among postmenopausal breast cancer patients has been found [58]. We observed that women with postmenopausal breast cancer who were MHT non-users and had an unfavorable lifestyle, including being overweight, were likely to have larger tumors compared with women with a favorable lifestyle. However, larger tumor size at diagnosis among postmenopausal breast cancer patients was not translated into shortened breast cancer-specific survival in our study. The association between an overall unfavorable lifestyle and higher overall mortality risk may reflect that an unfavorable lifestyle is often associated with other comorbid conditions [59, 60]. Consequently, breast cancer patients, even if they are cured of their cancer, may experience a reduced overall life expectancy due to pre-existing susceptibility to comorbidities and late adverse effects of treatment [61, 62]. This may, in particular, play a role for young breast cancer patients, as they tend to present with more aggressive tumor characteristics [63], which requires more comprehensive treatment, putting these women at risk of developing more severe treatment-induced chronic adverse effects [64]. Hence, the potential long period of post-diagnostic survival for most breast cancer patients provides a context in which long-term exposure to an unfavorable lifestyle may have a substantial impact on morbidity and overall mortality. Of note, we observed, among women with postmenopausal breast cancer, an inverse association between pre-diagnostic unfavorable lifestyle and breast cancer mortality. This observation may reflect that postmenopausal women with an unfavorable lifestyle are prone to die of causes other than their breast cancer [65].

The exact biological mechanisms explaining the association between lifestyle and breast cancer development have yet to be established, but an unfavorable lifestyle may accelerate aging and increase tumor growth, and result in chronic inflammation, metabolic dysfunction, and DNA methylation [66–68]. Telomere length has been proposed as a biomarker of biological age and a risk factor for cancer [69]. The unfavorable lifestyle factors included in our study have been independently observed to shorten telomere length, leading to accelerated aging [70–73], and potentially influence breast cancer prognosis [74]. An association between telomere length and breast cancer risk has shown conflicting results [75, 76]. However, one may propose that a healthy lifestyle may reduce the pace of telomere shortening, and delay the onset of age-related diseases, including breast cancer. In addition, high elevated levels of advanced glycation end products, observed in ER-positive breast cancers, have been associated with physical inactivity, tumor growth, and therapy resistance [77].

Our study had several strengths, which include its population-based approach, high attendance rate, the use of a standardized protocol for data collection, and measured height and weight. The study had high completeness rates of identification of breast cancer cases (Cancer Registry of Norway), and identification of death and emigration (Cause of death Registry). In an evaluation of the data quality of the Cancer Registry of Norway, completeness of reporting was estimated to be very high, 98.8% [31], limiting the possibility for any misclassification related to identification of cases. Thus, it is less likely that loss to follow-up or inadequate reporting influences our risk estimate, further reducing the chance of biased observations. Moreover, to lessen the chance that undiagnosed cancer could influence our results, we limited our study population to women who had no history of previous breast or other cancer within the first year after study inclusion. All medical records for the breast cancer patients were carefully reviewed by trained physicians, and included systematic abstraction of histopathology, oncological treatment, and recurrence and death [27]. The study also has several limitations. We did not have data on age at first pregnancy, family history of disease, weight at young adulthood, and adult weight gain. Moreover, the lifestyle factors and other baseline variables, except menopausal hormone therapy use, were based on a single pre-diagnostic measure collected average 20.1 years before censored. Thus, we were therefore unable to account for longitudinal changes in these factors on breast cancer risk and survival. However, tracking studies have shown that women tend to follow a trajectory of BMI, physical activity, blood pressure, and alcohol intake [78–80], suggesting an accumulated lifetime exposure. The sample size narrowed our possibility of performing further stratified analysis by breast cancer subtypes.

In conclusion, our study supports a dose–response association between lifestyle behavior and sporadic breast cancer. An unfavorable lifestyle may alter biological pathways, accelerating tumor growth, and thereby increase breast cancer risk, lower the age at onset of sporadic breast cancer, and shorten the life expectancy among breast cancer patients. Consequently, our results not only support a key role of a healthy lifestyle to improve breast cancer prevention, but also suggest that tailored personal lifestyle advice should be included in daily clinical practice of potential importance for treatment outcomes and life expectancy for women diagnosed with breast cancer.

## Electronic supplementary material

Below is the link to the electronic supplementary material.

Supplementary file1 (PDF 308 kb)

Supplementary file2 (PDF 152 kb)

Supplementary file3 (PDF 153 kb)

Supplementary file4 (PDF 142 kb)
