# Supplementary figures and images for: Exploring the effects of lifestyle on breast cancer risk, age at diagnosis, and survival: the EBBA-Life study
Source: Breast Cancer Res Treat. 2020 May 20;182(1):215–27. doi: 10.1007/s10549-020-05679-2 (PMC7275030; doi:10.1007/s10549-020-05679-2)

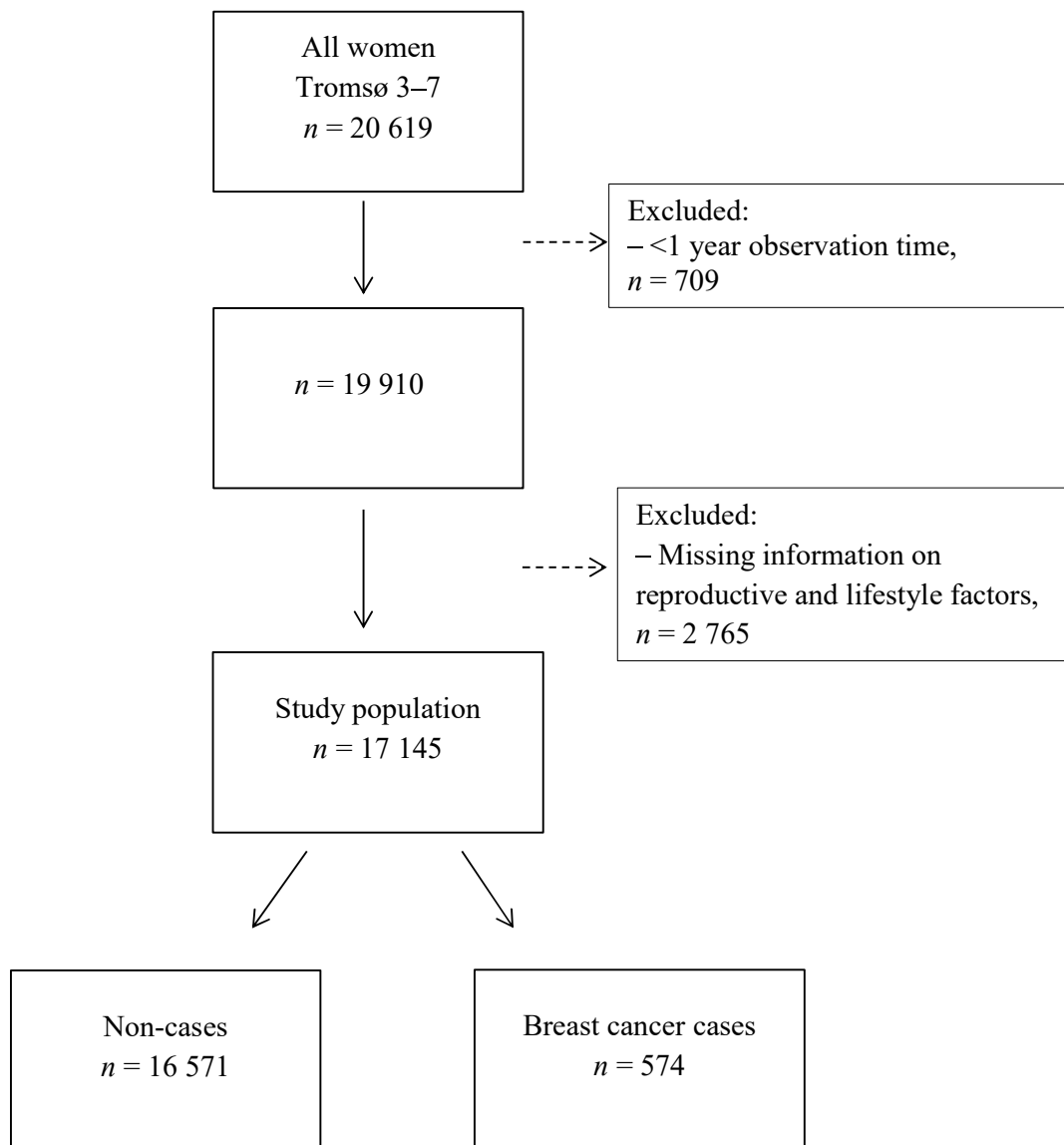

**Online Resource 1** Flow diagram for EBBA-Life study

Supplement: Supplementary file 1 — Supplementary file1 (PDF 308 kb) [file 10549_2020_5679_MOESM1_ESM.pdf]
