# Supplementary material for: Exploring the effects of lifestyle on breast cancer risk, age at diagnosis, and survival: the EBBA-Life study
Source: Breast Cancer Res Treat. 2020 May 20;182(1):215–27. doi: 10.1007/s10549-020-05679-2 (PMC7275030; doi:10.1007/s10549-020-05679-2)
Supplement: Supplementary file 2 — Supplementary file2 (PDF 152 kb) [file 10549_2020_5679_MOESM2_ESM.pdf]

Online Resource 2 Multivariable adjusted hazard ratios between lifestyle and incident breast cancer by breast cancer molecular subtype

	**ER-positive breast cancer**		**HER2-positive breast cancer**		**Triple negative breast cancer**	
	n = 362	HR (95% CI)	*n* = 85	HR (95% CI)	*n* = 75	HR (95% CI)
Number of unfavorable lifestyle factors[a]						
0 (ref)	39	1.00	24	1.00	20	1.00
1	118	1.27 (0.88–1.82)	32	0.80 (0.47–1.36)	33	0.95 (0.54–1.66)
2	115	1.30 (0.90–1.87)	21	1.00 (0.55–1.80)	15	0.73 (0.37–1.44)
3–5	90	1.43 (0.97–2.10)	8	0.98 (0.42–2.33)	7	0.70 (0.27–1.79)
P_{trend}		0.096		0.959		0.301

Cox's proportional hazard model.
[a] Unfavorable lifestyle factors: overweight, physical inactivity, alcohol use, smoking, and hypertension.
Adjusted to age (continuous), menopausal hormone therapy use (categorical), age at menarche (continuous), number of children (continuous),

Abbreviations: ER, estrogen receptor; HER2, human epidermal growth factor reseceptor-2; *n*, number of cases
