# Supplementary material for: Exploring the effects of lifestyle on breast cancer risk, age at diagnosis, and survival: the EBBA-Life study
Source: Breast Cancer Res Treat. 2020 May 20;182(1):215–27. doi: 10.1007/s10549-020-05679-2 (PMC7275030; doi:10.1007/s10549-020-05679-2)
Supplement: Supplementary file 3 — Supplementary file3 (PDF 153 kb) [file 10549_2020_5679_MOESM3_ESM.pdf]

Online Resource 3 Age-adjusted hazard ratios between lifestyle and incident estrogen receptor-positive breast cancer by menopausal status at diagnosis

	**Premenopausal ER-positive breast cancer**		**Postmenopausal ER-positive breast cancer**			
			**MHT non-users**		**MHT users**	
	n = 144	HR (95% CI)	*n* = 160	HR (95% CI)	*n* = 58	HR (95% CI)
Number of unfavorable lifestyle factors[a]						
0 (ref)	20	1.00	12	1.00	7	1.00
1	52	1.03 (0.62–1.74)	45	1.60 (0.85–3.02)	21	1.37 (0.62–3.03)
2	47	0.92 (0.55–1.56)	51	1.89 (1.01–3.56)	17	1.45 (0.64–3.26)
3–5	25	0.87 (0.48–1.57)	52	2.42 (1.27–4.63)	13	1.61 (0.67–3.85)
P_{trend}		0.475		0.004		0.312

Multivariable Cox's proportional hazard regression models.
[a] Unfavorable lifestyle factors: overweight, physical inactivity, alcohol use, smoking, and hypertension.
Adjusted to age (continuous), age at menarche (continuous), number of children (continuous),

Abbreviations: CI, confidence interval; ER, estrogen receptor; HR, hazard ratio; MHT, menopausal hormone therapy; *n*, number of cases; ref, reference
