# Supplementary material for: Exploring the effects of lifestyle on breast cancer risk, age at diagnosis, and survival: the EBBA-Life study
Source: Breast Cancer Res Treat. 2020 May 20;182(1):215–27. doi: 10.1007/s10549-020-05679-2 (PMC7275030; doi:10.1007/s10549-020-05679-2)
Supplement: Supplementary file 4 — Supplementary file4 (PDF 142 kb) [file 10549_2020_5679_MOESM4_ESM.pdf]

Online Resource 4 Tumor characteristics[a] of postmenopausal breast cancer cases (MHT non-users)

	Number of unfavorable lifestyle factors				
	**0 (*n*=17)** Mean (SD)/%	**1 (*n*=71)** Mean (SD)/%	**2 (*n*=72)** Mean (SD)/%	**3–5 (*n*=77)** Mean (SD)/%	*P*
**Tumor characteristics**					
Tumor size, mm	15.9 (12.3)	19.2 (11.9)	22.6 (17.0)	31.9 (28.5)	0.022
Number of metastatic lymph nodes	1.67 (1.97)	1.10 (2.27)	2.65 (4.64)	0.09 (0.30)	0.108
ER+, %	75.0	78.1	81.5	89.9	0.706
PgR+, %	43.8	58.1	60.1	50.0	0.543
HER2+, %	6.3	16.1	16.5	23.5	0.609
Ki-67	29.1 (17.5)	19.2 (17.4)	19.9 (16.4)	16.5 (14.2)	0.237
Histological grade	2.06 (0.70)	1.90 (0.73)	1.96 (0.70)	1.83 (0.74)	0.771

One-way ANOVA.
[s]Numbers may vary due to missing information.

Abbreviations: ER, estrogen receptor; HER2, human epidermal growth factor receptor-2; MHT, menopausal hormone therapy; *n*, number of cases; PgR, progesterone receptor; SD, standard deviation.
